# mTORC1-S6K Activation by Endotoxin Contributes to Cytokine Up-Regulation and Early Lethality in Animals

**DOI:** 10.1371/journal.pone.0014399

**Published:** 2010-12-21

**Authors:** Po-Shun Lee, Anna S. K. Wilhelmson, Anton P. Hubner, Samuel B. Reynolds, Dana A. Gallacchi, Terry T. Chiou, David J. Kwiatkowski

**Affiliations:** 1 Translational Medicine Division, Brigham & Women's Hospital, Boston, Massachusetts, United States of America; 2 Harvard Medical School, Boston, Massachusetts, United States of America; 3 Wallenberg Laboratory for Cardiovascular Research, University of Gothenburg, Gothenburg, Sweden; 4 Sahlgrenska Academy, University of Gothenburg, Gothenburg, Sweden; University of California Los Angeles, United States of America

## Abstract

**Background:**

mTORC1 (mammalian target of rapamycin complex 1) activation has been demonstrated in response to endotoxin challenge, but the mechanism and significance are unclear. We investigated the effect of mTORC1 suppression in an animal model of endotoxemia and in a cellular model of endotoxin signaling.

**Methodology/Principal Findings:**

Mice were treated with the mTORC1 inhibitor rapamycin or vehicle prior to lethal endotoxin challenge. Mortality and cytokine levels were assessed. Cultured macrophage-like cells were challenged with endotoxin with or without inhibitors of various pathways known to be upstream of mTORC1. Activated pathways, including downstream S6K pathway, were assessed by immunoblots. We found that mTORC1-S6K suppression by rapamycin delayed mortality of mice challenged with lethal endotoxin, and was associated with dampened circulating levels of VEGF, IL-1β, IFN-γ and IL-5. Furthermore, in vitro cellular studies demonstrated that LPS (lipopolysaccharide) activation of mTORC1-S6K still occurs in the presence of PI3K-Akt inhibition alone, but can be suppressed by concurrent inhibition of PI3K-Akt and MEK-ERK pathways.

**Conclusions/Significance:**

We conclude that cellular activation of mTORC1-S6K contributes to cytokine up-regulation and mortality in response to endotoxin, and may occur via multiple pathways.

## Introduction

Mammalian target of rapamycin (mTOR) is a serine/threonine kinase that regulates protein production essential for cellular proliferation. mTOR functions in two distinct complexes: mTOR complex 1 (mTORC1) and mTOR complex 2 (mTORC2). mTORC1 phosphorylates p70S6K (S6K) and 4EBP-1, leading to translational activation, ribosome biogenesis, and metabolic responses partly due to ribosomal protein S6 and eIF4E. mTORC1 is regulated by several interacting proteins, most significantly by levels of rheb-GTP, which are in turn regulated by the GTPase activating (GAP) activity of the TSC1/TSC2 complex. TSC1/TSC2 receives input signals from PI3K-Akt, WNT-GSK3, AMPK, and MAPK pathways and others, through phosphorylation events that reflect cellular energy stress, nutrient availability, and growth factor activation[1], [2]. Because of its essential role in cellular proliferation and frequent deregulation in cancer, mTOR has been intensively studied, leading to the use of rapamycin, a highly specific and potent mTORC1 inhibitor [3], in various forms of cancer[1], [4]. The role of mTOR in innate immunity has also begun to receive attention. It's been recognized that lipopolysaccharide (LPS) activates mTORC1 in various types of cells, such as neutrophils, monocytes, macrophages and dendritic cells[5], [6], [7], [8], presumably via PI3K-Akt pathway[2], [9], [10]. In addition, a porcine model of endotoxemia demonstrated mTORC1 activation in the liver but not muscle[11]. However the precise mechanism and significance of mTORC1 activation are not completely understood. For example, mTORC1 inhibition appears to be beneficial in one animal model of systemic inflammation[8], while in another animal model, there appears to be a detrimental effect from rapamycin treatment[7]. We report here that treatment of mice with endotoxin leads to mTORC1-dependent up-regulation of specific cytokines contributing to early endotoxemic lethality, and that LPS activates mTORC1 independent of PI3K-Akt pathway. Our data suggest mTORC1 may be a modifiable target in systemic inflammatory response.

## Materials and Methods

### Murine endotoxemia and rapamycin preparation

Wild type C57BL/6 male (Charles River Laboratories, Wilmington, MA), TNF-α receptor-null mutant (p55 and p75 double TNFR knockout) male mice[12] (the Jackson Laboratory, Bar Harbor, ME) had free access to a standard feed and water, and the Harvard Medical Area Standing Committee on Animals approved all procedures described (ARCH Protocol #07-09-1485R), according to standards as set forth in The Guide for the Care and Use of Laboratory Animals.

Rapamycin and vehicle were prepared as described previously [13], [14]. Briefly, rapamycin (LC Laboratories, Woburn, MA) dissolved at 20 mg/ml in ethanol was diluted in 0.25% Tween 80, 0.25% polyethylene glycol 400 (0.5–1.5 mg/ml). We had previously shown that rapamycin given at 6 mg/kg i.p. in mice is sufficient to maintain plasma rapamycin levels within the therapeutic ranges for at least 48 hr [13]. Eighteen 8–12 week old male C57BL/6 mice each weighing 18–20 g were injected i.p. with 25 mg/kg LPS (*Escherichia coli* O55:B5, Sigma, St. Louis, MO) and immediately resuscitated with 1 ml s.c. injections of PBS. Treatment with vehicle (9 mice) or 6 mg/kg rapamycin (9 mice) i.p. was given 30 min prior to LPS challenge. The mice were anesthetized for plasma and organ collections and then euthanized at 2, 6, and 24 hr (3 mice per group sacrifice at each time point) post LPS challenge. In addition, mice without LPS challenge served as controls. In a separate mortality experiment, mice received the same LPS challenge and were allocated to receive vehicle (10 mice) or rapamycin (6 mg/kg) (9 mice) treatment prior to LPS as described. The animals were monitored frequently, and survival was recorded for 5 days. Surviving mice were euthanized.

### Cytokine measurements

Plasma GM-CSF, IFN-γ, IL-1β, IL-2, IL-5, IL-6, IL-10, IL-12 and TNF-α cytokines were measured using a multiplex immunoassay assay (Millipore, Billerica, MA). The lower range of the assay is <3.2 pg/ml for each cytokine, and levels <3.2 pg/ml were assigned a value of zero. VEGF (vascular endothelial growth factor) was assayed using a commercial ELISA kit (R&D Systems, Minneapolis, MN) as directed.

### Organ protein extraction

Organs extracted from mice were mechanically homogenized in RIPA buffer (Boston Bioproducts, Boston, MA) supplemented with protease inhibitor cocktail (Roche, Indianapolis, IN) and a phosphatase inhibitor (Thermo Scientific, Waltham, MA), and incubated on ice for 30 min before centrifugation to remove insoluble tissue debris. Supernatant extracts were assayed for protein concentration by Bradford assay (BioRad Laboratories Inc. Hercules, CA) and normalized before immunoblotting.

### Cell Culture

Wortmannin, LY294002, and U0126 were purchased from Cell Signaling, Danvers, MA. Unless otherwise specified, cell culture media and supplements were from Invitrogen, Carlsbad, CA. Murine macrophage-like cells, RAW 264.7(ATCC, Manassas, VA, USA) were maintained in DMEM (Dulbecco's Modified Eagle's Medium) supplemented with 10% FBS and 1% penicillin-streptomycin-amphotericin B (PSA), in an incubator at 37°C in 5% CO_2_, until confluent. Primary murine peritoneal macrophages were harvested by peritoneal lavage of wild type C57BL/6 mice 72 hr after injection of 1.5 ml 2% thioglycolate media (Sigma, St Louis, MO). Primary cells from peritoneal lavage were placed on culture dishes in DMEM with10%FBS and 1%PSA, at 37°C, 5% CO_2_, and washed daily with fresh media to remove non-adherent cells for at least 5 days before experiments. Tsc2-null murine embryonic fibroblasts (MEFs) have been previously described from our lab[15]. The cells were seeded on 6-well plates at 500,000 cells/ml 24 hr prior to experiments. Inhibitors were added 30 min prior to LPS (100 ng/ml), and cells were harvested 2 hr post LPS.

### RNA Interference Studies

Small-interfering RNA (siRNA) constructs were purchased from Ambion (Austin, TX), and used as instructed by the manufacturer. Briefly, 30 nM (IKKβ) or 80 nM (raptor) of Silencer siRNA constructs against IKKβ (s68175), raptor (s92712) or nonsense negative control of corresponding concentration were incubated in Opti-MEM (Invitrogen, Carlsbad, CA) with NeoFX (Ambion) or RNAiMAX (Invitrogen) transfection agent. The mixture was then plated into 6 or 12-well plates and overlaid with 1.5×10^5^ cells/ml in Opti-MEM for 24 hr. Media was replaced with fresh DMEM with 10%FBS, 1%PSA and incubated for another 24 hr before LPS challenge.

### Western blotting

Protein samples were analyzed by SDS-PAGE using 4–12% NuPAGE Gel (Invitrogen), and transferred to a nitrocellulose membrane. Immunoblotting was performed by standard methods using HRP-conjugated secondary antibodies, and chemiluminescence using Supersignal West Pico Chemilumincesent substrate (Thermo Fisher Scientific, Pittsburgh, PA) and exposure to film. All antibodies, except anti-tubulin (Abcam, Cambridge, MA) were purchased from Cell Signaling.

### Statistics

Statistical analyses were performed using Prism 4 (Graphpad Software, La Jolla, CA). Survival curves were plotted using the method of Kaplan and Meier and analyzed using the logrank test. Temporal cytokine levels between 2 groups were compared by Repeated Measures ANOVA. A p-value<0.05 was considered significant.

## Results

**Figure 1:**
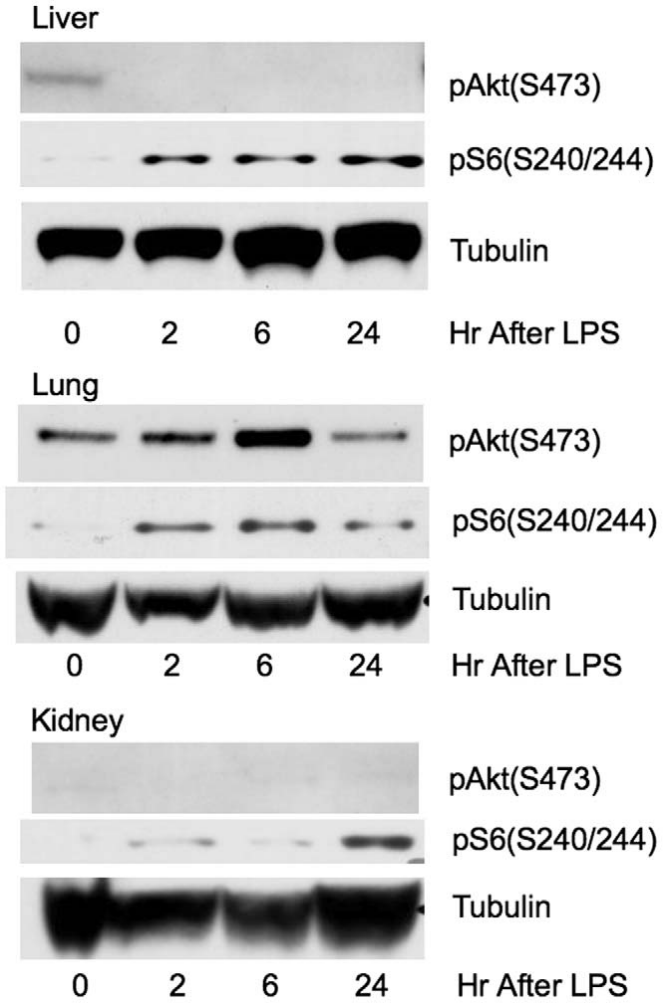
mTORC1-S6K up-regulation in the livers, lungs, and kidneys of mice treated with endotoxin. Representative immunoblots of normalized organ protein extracts probed for pS6 (S240/244), a downstream target of mTORC1-S6K are shown here. Mice were subjected to 25 mg/kg of LPS i.p. and sacrificed at 2,6, and 24 hr later. Untreated mice served as time 0 hr controls. mTORC1-S6K activation could be seen as early as 2 hr after LPS challenge in the livers and lungs of endotoxemic mice. mTORC1-S6K was also activated in the kidneys at 24 hr post challenge. Increased pAkt (S473) was seen only in the lung extracts post LPS challenged, occurring at 6 hr post LPS, after mTORC1-S6K activation, and was not seen in either liver or kidney.

mTORC1-S6K was highly activated in lungs, kidneys, and livers of mice treated with LPS, as assessed by an increase in phosphorylated S6(S240/244) (pS6,S240/244). This occurred within 2 hr of LPS challenge in the liver and lung, and persisted for at least 24 hr (Figure 1). PI3K-Akt activation (pAkt,S473), however, could only be demonstrated in the lungs after mTORC1-S6K activation, 6 hr post LPS challenge. These data suggest that mTORC1-S6K up-regulation in organs of endotoxemic mice may occur without PI3K-Akt activation.

**Figure 2:**
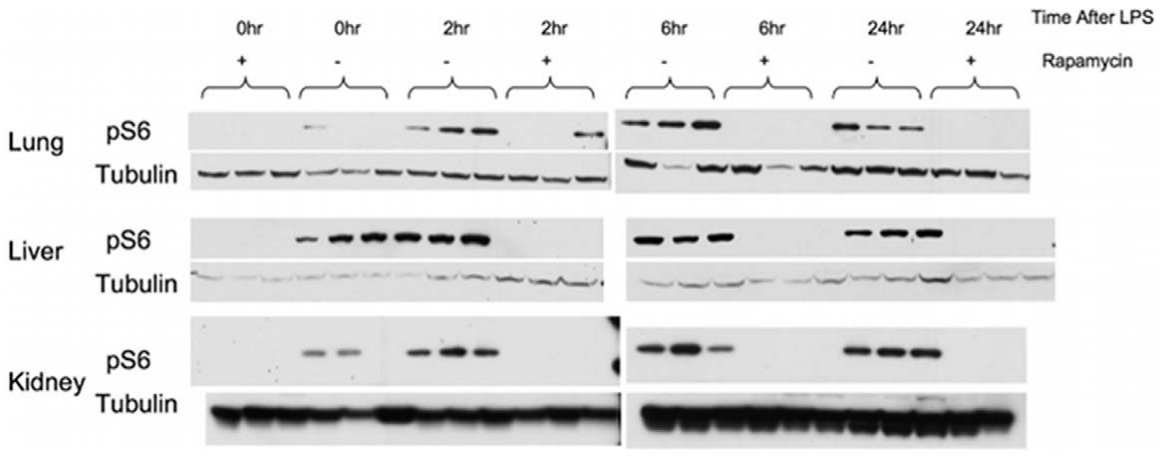
Rapamycin treatment effectively suppressed mTORC1-S6K signaling. Following LPS challenge in vivo, mice were treated with vehicle or 6 mg/kg rapamycin 30 min prior to 25 mg/kg LPS challenge. Organs were harvested from mice at 2, 6, 24 hr post challenge. Immunoblots of organs extracted from 3 mice from each treatment group probed for pS6 (S240/244), a downstream target of mTORC1-S6K are shown here. Unchallenged mice with or without rapamycin served as controls (time 0 hr). Rapamycin treatment successfully suppressed mTORC1-S6K up-regulation in endotoxemic mice as reflected by the near-absent pS6 signals.

**Figure 3:**
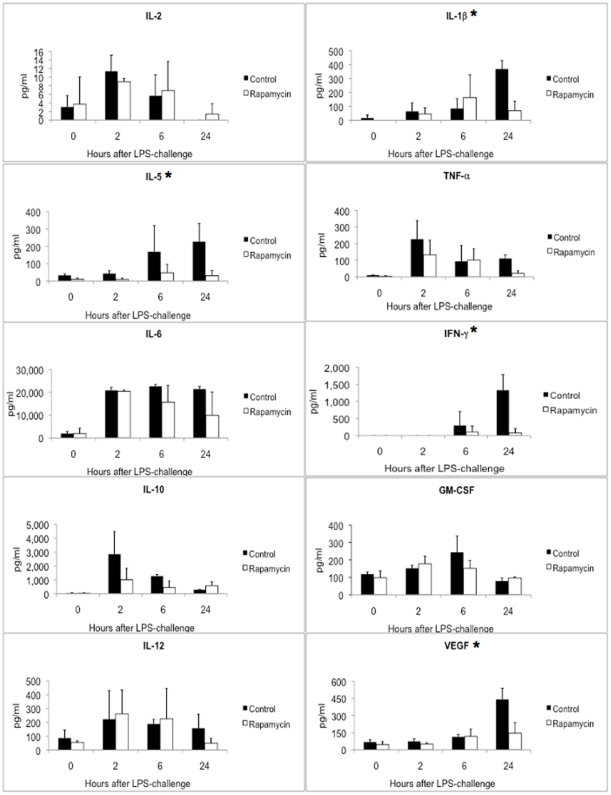
Rapamycin suppression of mTORC1-S6K was associated with lower cytokine levels. Following LPS treatment in vivo, circulating cytokine levels of LPS-challenged mice pre-treated with vehicle (solid bar) or rapamycin (open bar) at 0 (no LPS), 2, 6, and 24 hr post LPS are shown here. Data are expressed as mean of three mice in each group at each time point, with error bars representing standard deviations. Statistical analysis was performed using Repeated Measures ANOVA, and p values<0.05 are considered as significant (*). mTORC1-S6K suppression by rapamycin led to decreased circulation levels of IL1-β, VEGF, IFN-γ and IL-5. There was a trend toward lower TNF-α levels with a p value of 0.058.

**Figure 4:**
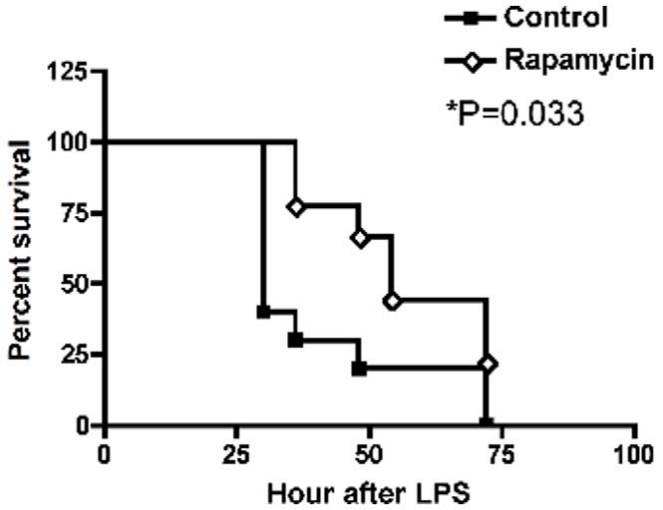
Rapamycin suppression of mTORC1-S6K enhanced early survival of endotoxemic mice. Kaplan-Meier curves of LPS-challenged mice treated with vehicle control (N = 10) or rapamycin (N = 9) are shown here. Rapamycin-treated mice had significantly better survival compared to vehicle-treated (P = 0.033, logrank test). However, all mice had died by 72 hr after LPS challenge.

Since mTORC1-S6K was highly activated by LPS treatment, we explored the effects of rapamycin treatment on this activation and other events occurring during the subsequent endotoxic shock in these mice. Rapamycin (6 mg/kg) administration 30 min before LPS effectively suppressed mTOC1-S6K activation in all tissues (Figure 2). In addition, cytokine profiling demonstrated that rapamycin pre-treatment led to significant down-regulation in the expression of several cytokines: IL-1β, IL-5, IFN-γ, and VEGF as shown in Figure 3. The suppressive effect by rapamycin treatment is especially apparent 24 hr post LPS treatment. In contrast, levels of IL-2, IL-6, IL10, IL-12, and GM-CSF showed no significant change while TNF-α showed a trend toward lower levels with rapamycin treatment (P = 0.058). These effects correlated with a transient improvement in mortality in these mice following lethal endotoxin injection (Figure 4). However, notably, this single pre-LPS dose of rapamycin did not prevent late mortality. Our data are in contrast with a previous report of rapamycin enhancing IL-1β production in endotoxemic animals[7] and more consistent with a recent report of lower pro-inflammatory TNF-α and IL-6 in response to LPS by rapamycin treatment[5]. We also did not observe a significant alteration of IL-10/IL-12 induction following LPS with rapamycin treatment, in contrast to previous reports on cultured cells treated with LPS[6], [8].

**Figure 5:**
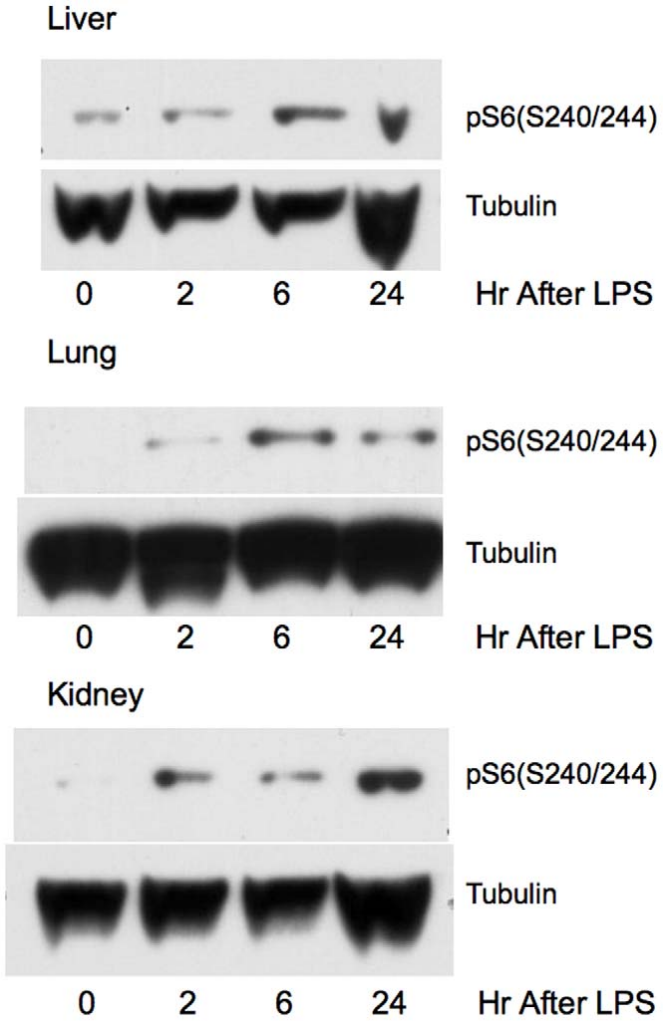
mTORC1-S6K activation in endotoxemic TNFR-null mice. Transgenic mice deficient in TNF-α receptors (TNFR) were given LPS and sacrificed at 2, 6, and 24 hr later for organ extraction. Representative immunoblots probed for pS6 (S240/244), a downstream target of mTORC1-S6K are shown here. mTORC1-S6K activation could be observed in the livers, lungs and kidneys of these mice similar to wildtype mice.

TNF-α is thought to activate mTORC1 through IKKβ phosphorylation and inactivation of TSC1[16]. This is a potential mechanism by which LPS treatment leads to mTORC1-S6K activation in LPS-challenged animals, independent of PI3K-Akt pathway. However, mTORC1-S6K activation occurred equally well in mice deficient for TNF-α receptor knockout, suggesting TNF-α signaling does not play a significant role in mTORC1-S6K activation in endotoxemia (Figure 5). It is important to note that without careful comparisons of the degrees of mTORC1-S6K activation between wild type and TNFR knockout mice, we could not rule out the possibility of TNF-α playing a minor role in LPS induction of mTORC1-S6K.

**Figure 6:**
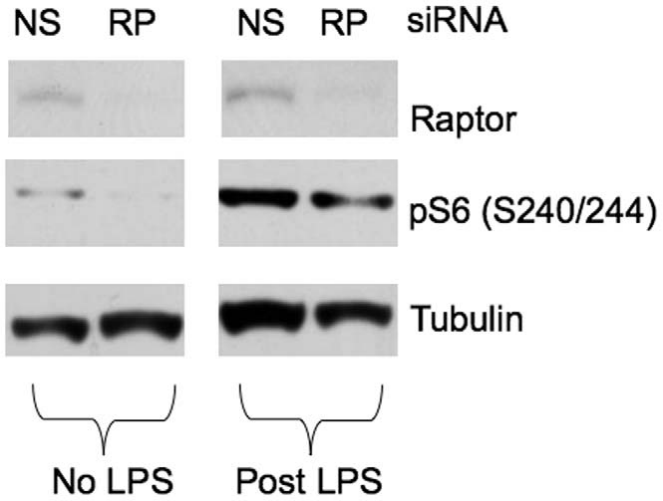
LPS activation of S6K was raptor dependent. RAW 264.7 cells were subjected to siRNA knockdown of raptor (RP), an essential component of mTORC1 or nonsense control (NS). Cells were then challenged with or without 100 ng/ml of LPS for 1 hr. Representative immunoblots shown here demonstrated the blunted LPS activation of S6K, as reflected by pS6 (S240/244) in cells subjected to raptor knockdown, supporting that mTORC1 activation by LPS led to S6K activation.

We also examined the effects of LPS treatment on macrophages using RAW 264.7 murine macrophage-like cells. Knockdown of raptor, an essential component of mTORC1, by siRNA blunted LPS-induced increase in S6 phosphorylation at S240/244 (Figure 6) confirming that mTORC1 is activated by LPS leading to S6K phosphorylation of S6.

**Figure 7:**
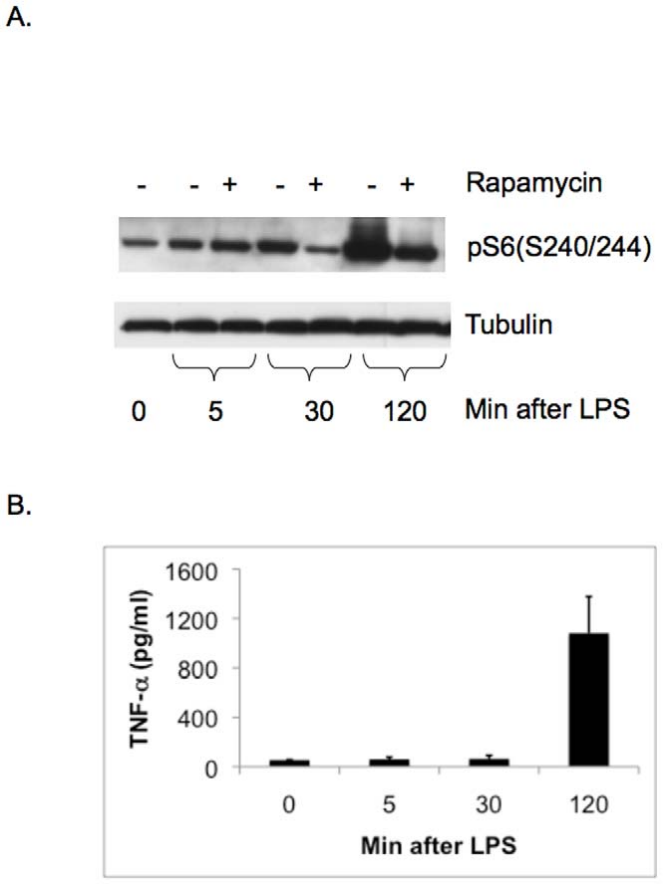
TNF-α secretion of LPS-treated macrophages occurred after mTORC1-S6K activation. Conditioned media and cell lysates of RAW cells treated with 100 ng/ml of LPS were collected at various time points. The immunoblots probing for pS6(S240/244) (A.), and the averages of TNF-α levels in the conditioned media from triplicate experiments with error bars representing standard deviations (B.) are shown here. mTORC1-S6K activation occurred as soon as 30 min after LPS when TNF-α levels were not detectably increased at that time point.

Rapamycin sensitive LPS activation of mTORC1-S6K in RAW cells occurred within 30 min of LPS challenge (Figure 7A), while TNF-α levels were not detectably increased in the conditioned media at that time point (Figure 7B). Therefore, it appears that TNF-α appears is not required for mTORC1 activation response in LPS-treated RAW cells.

**Figure 8:**
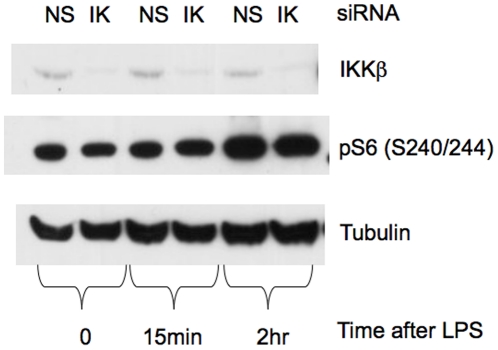
IKKβ was not required for LPS-induced mTORC1-S6K activation. IKKβ was suppressed in RAW cells by siRNA (IK) 48 hr prior to LPS stimulation. Nonsense siRNA constructs (NS) served as controls. Representative immunoblots show that IKKβ suppression had no effect on mTORC1-S6K activation in LPS-treated RAW cells as reflected by pS6 levels.

mTOR shares structural similarities with PI3Ks, and classic “specific PI3K inhibitors” such as wortmannin and LY294002 also can inhibit mTOR function when given at high doses[17], [18]. After preliminary studies examining dose-response characteristics, we found that wortmannin (100 nM) and LY294002 (12.5 µM) effectively suppressed PI3K-Akt signaling in response to LPS, as assessed by phosphorylation of Akt at the S473 site, but failed to inhibit mTORC1-S6K activation (Figure 8). Thus, LPS activation of mTORC1 appears to be independent of PI3K pathway, correlating with data from endotoxemic mice.

**Figure 9:**
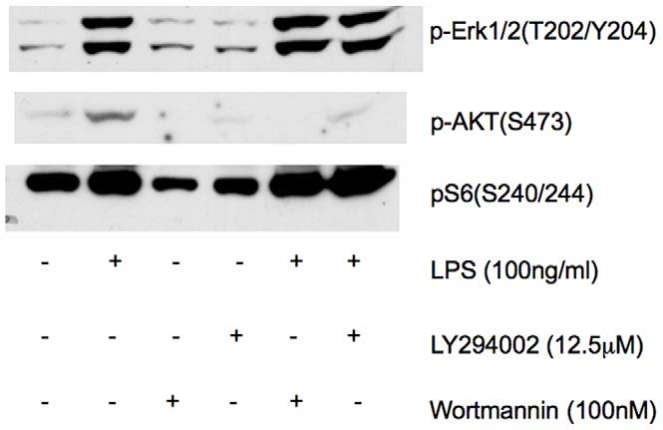
PI3K-Akt inhibition did not effect LPS-induced mTORC1-S6K activation. RAW cells pre-treated with 100 nM wortmannin, 12.5 µM LY294002, or vehicle control prior to 100 ng/ml LPS challenge were collected 2 hr post LPS. Representative immunoblots demonstrating successful suppression of p-Akt with wortmannin or LY294002 did not affect mTORC1-S6K activation as assessed by pS6 levels. Activation of ERK was also not affected by these compounds.

Since IKKβ can suppress TSC1 leading to mTORC1 activation [16], and LPS can activate IKKβ directly [19], we asked whether LPS activates mTORC1 via IKKβ. Using siRNA, Figure 9 shows that IKKβ knockdown had no effect on LPS-induced mTORC1-S6K activation in RAW cells, suggesting IKKβ is also not an essential component of LPS-induced mTORC1-S6K activation.

**Figure 10:**
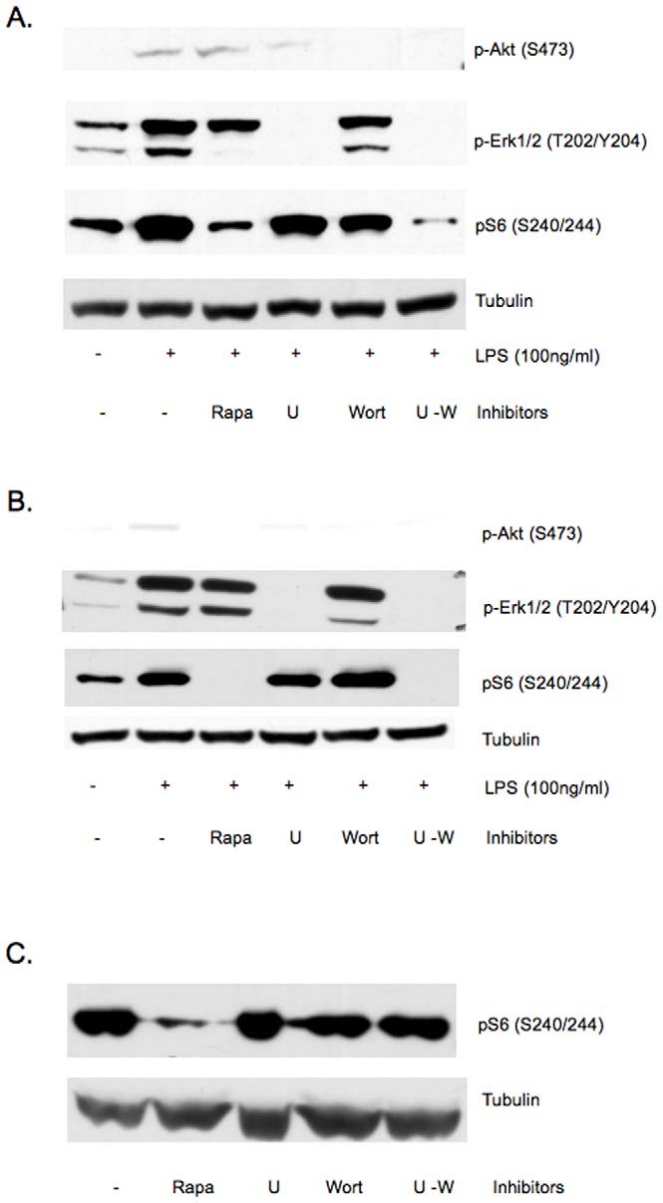
Concurrent inhibition of PI3K and MEK-ERK pathways suppresses LPS-induced mTORC1-S6K activation in macrophages. Representative immunoblots of RAW cells (A) and murine peritoneal macrophages (B) pretreated with vehicle control, 20 nM rapamycin (Rapa), 5 µM MEK1/2 inhibitor U0216 (U), 100 nM wortmannin (Wort), or combination of U0216 and wortmannin (U–W) 30 min prior to LPS stimulation. Cells were harvested 2 hr post LPS. Only the combined Inhibition of MEK1/2-ERK and PI3K-Akt significantly suppressed mTORC1-S6K activation by LPS, as indicated by pS6 levels. The same doses of pharmacologic inhibitors were applied to Tsc2-null MEFs (C), and did not suppress mTORC1-S6K activity as reflected by pS6, suggesting the effects of these compounds are dependent upon Tsc2.

Because ERK (extracellular signal-regulated kinase) can phosphorylate and negatively regulate the GAP activity of TSC2 and activate mTORC1[20], we examined whether activation of ERK downstream of LPS is responsible for mTORC1-S6K activation. MEK inhibitor U0216 given at a dose that has no direct effect on mTORC1-S6K suppressed ERK phosphorylation but did not completely suppress mTORC1-S6K activation in LPS simulated RAW cells (Figure 10A). In addition, wortmannin completely suppressed p-Akt(S473), but did not completely abolished LPS-induced pS6 up-regulation. However, the combination of wortmannin and U0216 completely suppressed mTORC1-S6K activation by LPS, suggesting LPS may activate mTORC1-S6K via MEK-ERK or PI3K-AKT pathway, and neither pathway is required (Figure 10A). Similar experiments on primary murine peritoneal macrophages yielded same results (Figure 10B). We confirmed the lack of direct effect of these inhibitors on mTORC1 in Tsc2-null murine embryonic fibroblasts (MEFs). Figure 10C showed that mTORC1, which is constitutively active in Tsc2-null cells, was not suppressed by U0216 or wortmannin, either individually or in combination at the doses used in this study.

## Discussion

We have documented that mTORC1-S6K is activated in several organs of mice challenged with LPS, that mTORC1-S6K inhibition by rapamycin treatment leads to decrease in levels of several inflammatory cytokines, such as IL-1βand VEGF, and transient improved survival, and that mTORC1-S6K activation by LPS may occur with PI3K-Akt suppression. Because we have focused on the initial activation of mTORC1 by LPS, only one dose of rapamycin treatment was used in the animal mortality experiment. It is certainly possible that repeated doses of rapamycin may improve survival in this model. However we have previously shown in another mouse model that this single dose of rapamycin leads to persistent levels of rapamycin in serum and liver lysates, well above the conventional therapeutic range, for at least 48 hours [13], suggesting that there would be no major benefit to a second dose. Nevertheless, full evaluation of this possibility will require additional study.

Our data are in distinct contrast to the study by Schmitz et al, who reported that rapamycin treatment enhanced IL-1β levels and worsened survival in mice challenged with lethal dose of LPS[7]. However, several important differences between the two studies may explain the contradictory findings. Schmitz et al treated mice with a lower dose of rapamycin (1.5 mg/kg) compared to the 6 mg/kg dose used in our study. Unfortunately, Schmitz et al did not offer evidence of successful mTORC1 suppression in their study; therefore, it is possible that they did not effectively suppress mTORC1. In addition, Schmitz et al treated mice with daily rapamycin for 3 days prior to LPS challenge. It has been recognized that extended rapamycin treatment can lead to compensatory changes in the PI3K-Akt pathway[1], [21]. Pre-treatment with rapamycin in Schmitz et al's study might have enhanced PI3K-Akt response to endotoxin leading to further exaggerated inflammatory response. Finally, Schmitz et al tested female mice while we studied male mice; gender differences in LPS and rapamycin response may also explain different observations.

Our findings are more consistent with the study by Lorne et al who found rapamycin treatment suppressed pro-inflammatory cytokines elicited by LPS[5]. In addition, we found VEGF secretion is an mTORC1-S6K dependent process, similar to other reports in cancer-related studies[22], [23]. VEGF has been implicated to play a significant role in acute respiratory distress syndrome (ARDS) [24] and sepsis[25], and our data provide additional target to intervene in VEGF-dependent pathology. Therefore, mTORC1-S6K inhibition by rapamycin likely lessened endotoxemia death by suppressing pathologic levels of cytokines, such as IL-1β and VEGF.

Interestingly, rapamycin suppression of mTORC1-S6K also reduced circulating levels of IL-5 and IFN-γ. While mTOR has been implicated in the IFN-induced signaling[26], [27], our data suggest mTORC1-S6K activation plays a critical role in IFN-γ up-regulation induced by LPS. These data suggest the possibility that mTOR may have a previously unrecognized role in IFN-γ and IL-5 production.

We also report that LPS-induced mTORC1-S6K activation does not necessarily depend on PI3K-Akt pathway as previously believed[2], [9], [10]; rather, MEK-ERK may play a significant role serving as an alternative pathway for mTORC1-S6K activation by LPS. In tumor-like cells, ERK phosphorylates TSC2 leading to the dissociation of TSC1/TSC2 complex with subsequent up-regulation of mTORC1[20]. It is possible that the same pathway is involved in LPS activated inflammatory cells, such as macrophages. While the precise mechanism of how LPS-TLR4 activates MEK-ERK pathway remains incompletely understood, ERK activation appears to play a major role in cellular cytokine secretion in response to LPS[19]. Our data may point to mTORC1-S6K activation as a possible mechanism of ERK-dependent cytokine production in response to LPS.

In conclusion, we document the beneficial effect of mTORC1-S6K suppression in an animal model of endotoxemia, associated with down-regulation of several cytokines, including IL-1β and VEGF, thought to be important in acute inflammatory response. Furthermore, LPS can activate mTORC1-S6K with suppressed PI3K-Akt, via alternative MEK-ERK pathway. Our data provide new insight into LPS signaling and may support mTORC1 as a possible target for therapeutic intervention in inflammatory conditions.
